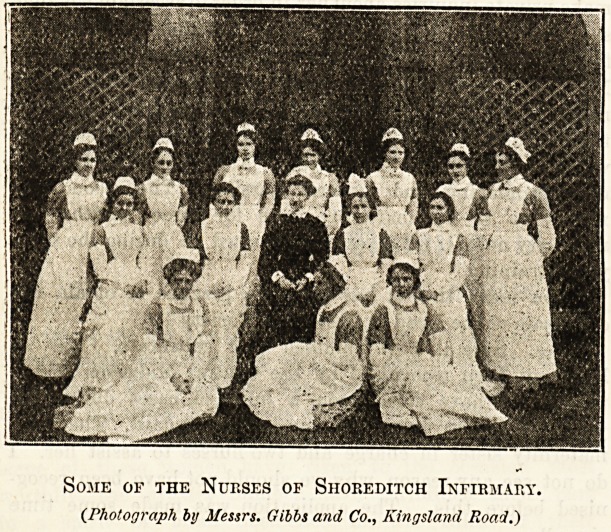# The Hospital. Nursing Section

**Published:** 1905-05-27

**Authors:** 


### The Hospital.

### Nursing Section.

Contributions for this Section of "The Hospital" should be addressed to the Editor, "The Hospital" Nursing Section, 28 & 29 Southampton Street, Strand, London, W.C.

No. 974.—Vol. XXXVIII. SATURDAY, MAY 27, 1905.

### Notes on News from the Nursing World.

### RESIGNATION OF MISS PETER.

Queen's Nurses throughout the country will learn with much regret that Miss Peter has resigned the post of general superintendent of Queen Victoria's Jubilee Institute, which she has held with conspicuous ability and tact, sparing no pains to promote the extension of the movement, and endearing herself personally to all who have had the pleasure of coming into contact with her. For the appointment which thus becomes vacant, applications are now invited. The salary is £250 at the outset, increasing to £300. Candidates should send their age, qualifications, with copies of testimonials, before June 20 to the Honorary Treasurer, 120 Victoria Street, S.W.

### CONFERENCE OF THE JUBILEE INSTITUTE COUNCIL.

The usual annual conference of the Council of Queen Victoria's Jubilee Institute and representatives of the affiliated County Nursing Associations took place last week, and a considerable number of members were present. The proceedings were private, but we understand that discussions of some length took place on school nursing, emergency nurses, the training of village nurses, and the methods of raising funds for County Nursing Associations. The last is one of practical importance, but we imagine that it must necessarily be left to the managers of each County Association to form their own plans for replenishing the exchequer according to local feeling and conditions.

### NURSING QUESTIONS IN PARLIAMENT.

It is, of course, desirable that any nursing question of real importance to the community should be brought before Parliament if it cannot be dealt with satisfactorily by any other means. But it is a matter for congratulation that the President of the Local Government Board is not subjected to such categories as Mr. Walter Long was expected to answer the other day in the House of Commons. Mr. Sloan wanted to know from the Irish Secretary how many nurses were acting as paid nurses in the Ballyshannon Union; was he aware that one of the nurses who was in charge left off duty on February 14 last and had not returned since; if so, would he say if she had sent in her resignation, and on what date; who was the nurse in charge at present, and in what date was she appointed? Mr. Long was able to definitely reply that three nuns are employed as paid nurses in the infirmary of the Ballyshannon Workhouse; but he confessed that he had no information to show that one of them had vacated her position either temporarily or permanently. Such points as these might surely be referred to the Local Government Board in Dublin.

### PRINCESS CHRISTIAN AND BADGES FOR NURSES.

It will be remembered that in our last issue Miss Dorothy A. Davey stated that Princess Christian approves of her proposed distinctive badge for nurses, and had commanded that the matter be placed before the Royal British Nurses' Association, of which she is President, at their next meeting. Miss Annie Hobbs, secretary of the Royal British Nurses' Association, observing that this seems liable to lead to misapprehension, sends us a copy of the letter which was sent, by command of Princess Christian, to Miss Davey through Mrs. Coster, the Nurse Honorary Secretary of the Association. She said: "I am commanded by her Royal Highness Princess Christian to acknowledge and thank you for your letter to her Royal Highness and to say that it shall be laid before the Executive Committee of the Royal British Nurses' Association at its meeting, May 19th." We need hardly say that it by no means follows that Princess Christian approves the proposal of Miss Davey.

### THE NECESSITY OF A PROPER DIETARY.

We commend to the attention of the Halifax Guardians, who have lately had under discussion the question of the dietary of the nurses at the Poor-law Hospital, the diet provided at the Shoreditch Infirmary. In more ways than one the Shoreditch Infirmary is in the van of progress, in so far as Poor-law institutions are concerned, and the details given by the matron to our Commissioner in the interview which is reported this week, show that the importance of providing not only adequate and well-cooked food for those who attend the sick, but also ample variety, is recognised by the Guardians. It is not merely essential, as a matter of humanity, that nurses who work hard should not be underfed; it is wise, as a matter of policy, that they should have a liberal diet. The stronger they are the better health they enjoy, the more likely they are not to be on the sick-list themselves, and the more fit to adequately discharge the most laborious of their duties with success.

### MATRONS OF COTTAGE HOSPITALS.

There were no fewer than 83 candidates for the office of the first matron of Walton-on-Thames, Hersham, and Oatlands Cottage Hospital, of whom six were selected. The names of these were submitted to the committee of management for decision, and they ultimately decided to appoint Miss Rosita Macandrew. The qualifications of Miss Macandrew are that she has had training, general and children's, at three of the leading London hospitals; that she has enjoyed considerable experience as a Queen's nurse, and has acted, temporarily, as matron of a cottage hospital and a surgical and convalescent home. She discharged her duties so satisfactorily in the latter capacity that she was recently asked to take the post of matron permanently. We rejoice that the choice of the committee has fallen upon a fully trained and thoroughly well equipped candidate. The work of cottage hospitals is sometimes described as comparatively unimportant, but it should not be so regarded. Very serious cases are often received, and care and attention in matters of detail are particularly needed on the part of the matron. She should, in fact, be an all-round nurse of the best type, capable of turning her hand to anything and of being equal to any emergency.

### THE NURSE AND THE MOTHERS.

At a meeting held last week in the interests of the Infants' Health Society, Dr. Vincent, of the Infants' Hospital, in explaining how it is proposed to endeavour to secure the supply of pure milk for infants in St. Pancras with the view of saving infant life and giving the infants of poor parents a chance of a sound constitution in after life, said that everything possible would be done by the depôt to encourage maternal nursing under proper conditions. The main feature of the contemplated arrangements is the appointment of an honorary physician and a trained nurse. The physician is to attend at the depôt to examine infants and order milk of a suitable character, and the nurse is to institute visiting in the homes to get in touch with the mothers. It is not intended to inaugurate the scheme until at least £200 has been promised, and the nurse will then be selected. Assuming that the necessary money is subscribed, a great deal will depend upon her ability to influence the mothers and convince them that it is of the utmost importance that their young children should be fed with pure milk.

### A MATRON'S AUTHORITY.

We do not think that there are many matrons who, while very properly making it a rule that the illness of a nurse should be reported by the sister under whom she is working, resent, as one of our correspondents resents, the idea that if a medical officer sees a nurse is indisposed he should order her off duty and request the sister to inform the matron of his action. It is quite conceivable that a nurse may be ill without complaining, or that the sister under whom she works may not be satisfied that she is indisposed, and if, in spite of absence of complaint or lack of perception, the medical man observes a nurse in the wards who is not, in his opinion, fit to be there, we hold that he ought at once to send her away. His intervention, however, by no means relieves the matron of her obligation in the matter, nor is it an abuse of her prerogative.

### INVALID COOKERY CLASSES AT WAKEFIELD.

The probationers of Clayton Hospital, Wakefield, who attended the matron's invalid cookery classes during the winter were examined last week, and the result was very encouraging. The chairman of the house committee presented two prizes. One for senior nurses was won by Nurse E. Ault, with honourable mention of Nurses L. Armstrong and S. Moore, and the other for junior nurses was obtained by Nurse M. E. Thompson, with honourable mention of Nurses E. Bradford and N. Boast. We wish that the admirable practice in force at the Wakefield Hospital were more generally observed. The importance of invalid cookery classes for probationers is still far from being sufficiently well recognised.

### PORTERESS AS MATRON.

The Glossop Corporation have appointed as matron of Gamesley Infectious Hospital the former porteress at several institutions. Her husband, who has also been porter at several hospitals, has been selected as caretaker. Supposing that the number of candidates was limited, we think that it is a distinct disadvantage that the wife of a caretaker should be the matron of a hospital, even if she has enjoyed the proper training of a nurse, and in this instance it does not appear that the requisite qualification is possessed.

### GUY'S HOSPITAL LADIES' ASSOCIATION.

The Countess of Bective presided on Tuesday over the tenth annual general meeting of the Guy's Hospital Ladies' Association. The meeting took place in the Court-room of the hospital, and was attended by more than 100 members, including the Countess of Selkirk, Mrs. Benyon, Mrs. Cosmo Bonsor, Lady Stevenson, and Lady Perry. Lady Bective congratulated the Association upon the progress it had made in 10 years, the membership having now reached 1,050, and there being 18 local branches. The clothing for the patients, contributed by members, amounting to over 2,000 garments, was on view, and subsequently those present at the meeting were able to inspect the wards of the hospital.

### DRAWING-ROOM MEETING AT BELTON HOUSE.

By permission of Countess Brownlow a meeting was held the other day in the library at Belton House, Grantham, to further the aims and objects of the Lincolnshire Nursing Association. Lady Brownlow herself presided, but confined her remarks to welcoming the company, and then called upon Miss Amy Hughes to deliver a speech. Miss Hughes having explained the work of the county associations affiliated to the Jubilee Institute, and paid a compliment to Lincolnshire as a pioneer county in the matter of the movement for the multiplication of village nurses, a brief address was delivered by Miss Glover, the county superintendent. Miss Glover stated that last autumn the Association opened five new districts, two employing fully-trained Queen's nurses, one a nurse with some prospect of further training, and two nurses who hold midwifery certificates. This year they had already had five districts applying for nurses, one of whom would be a Queen's nurse, and the others village nurses. As Miss Glover added that to do the work properly an income of £500 a year is needed, and that last year the amount raised was not more than £200, there is ample scope for the liberality of the Lincolnshire people.

### DEATH OF A LIVERPOOL MATRON.

We regret to learn of the death of Miss Edith Louise MacCarthy, who about six months ago resigned the post of matron of Parkhill Fever

Hospital, Liverpool, owing to ill-health. Miss MacCarthy was trained at Leeds General Infirmary, and on leaving that institution became sister at Hull Fever Hospital. Her next appointment was that of matron of St. Helen's Fever Hospital, and she was subsequently chosen for the responsible office of matron of Parkhill Fever Hospital. Her kindly manners and winning disposition endeared her to a large circle of friends both inside and outside the institution with which at different times she was connected, and the early termination of a busy and useful career is much to be deplored.

### NURSING AT NEWLYN.

In spite of its natural beauties and health-giving breezes, Newlyn finds plenty of work for a nurse to do. Last year the number of cases treated was 175, and the number of visits paid 4,646. It is noteworthy that no fewer than 41 patients suffered from poisoned hands and wounds on the legs. As to the financial position, the expenditure was only a trifle in excess of the receipts, but the latter, we regret to observe, from the report which was read at the annual meeting, were less by nearly £10 than those of the previous year. Bearing in mind the fact that Newlyn is a fashionable artistic centre, and that many persons of considerable means make prolonged stays in the picturesque village, profiting, no doubt, by their surroundings, we think that the Nursing Society should receive more liberal support than appears to be accorded to it.

### A SERVICE MEDAL.

The Committee of Gravesend Hospital have awarded a gold medal to Nurse Webb in commemoration of the completion of ten years' faithful service in connection with the institution. The complimentary remarks made by the Chairman in presenting the medal were endorsed by the Chairman of the Northfleet Diamond Jubilee Samaritan Fund, for which Miss Webb is made district nurse. This is the fourth occasion on which a gold medal has been given to a nurse at Gravesend Hospital, the expense being in each instance defrayed by the President.

### SUICIDE OF A SUPERANNUATED SUPERINTENDENT.

The death of Miss Eveth, who was superintendent nurse at Oldham Workhouse Infirmary for some time, is reported under the saddest conditions. In December 1903 Miss Eveth, who was 55 years of age, retired under the superannuation provisions, and for the last four months she had been out of health, and had been attended for nervous debility. A nurse, who formerly resided with her, went to see her a few days ago, and found her in one of the back bedrooms hanging from a bedpost by a silk necktie, life being quite extinct. In a letter in the pocket of the deceased she stated that she could no longer bear "this mental anguish," and at the inquest the jury returned a verdict of suicide whilst temporarily insane. The event has caused a painful sensation in Oldham, where Miss Eveth was well known and much respected.

### A NURSE'S STRUGGLE WITH A MADMAN.

An incident at Lincoln County Hospital last week emphasises the value of a nurse who combines physical courage with physical strength. Before Miss Lakum, the night superintendent, could intervene, a patient suddenly rose from his bed and threw himself through the window of the ward into the grounds, six feet below. Notwithstanding his injuries, he immediately jumped up, and endeavoured to make good his escape. The night superintendent, however, without hesitation, went in pursuit of the man, and overtaking him a quarter of a mile from the hospital she closed with him, maintaining her hold in spite of his desperate struggle to free himself, until a police constable came to her aid. The patient was then got back to the hospital, and after his wounds had been dressed he was conveyed to the lunatic asylum at Bracebridge Heath. But for the presence of mind and pluck of the night superintendent he would undoubtedly have either succeeded in killing himself or in doing grievous harm to someone else.

### THE SUPPLY OF MIDWIVES.

As there is very considerable difficulty in several parts of the country in obtaining fully-qualified midwives for district work among the poor we are glad to be able to state that the Association for Promoting the Training and Supply of Midwives are prepared to recommend competent women who have been trained under their auspices. In making application to the secretary at Dacre House, Dean Farrar Street, Westminster, S.W., it should be mentioned when the midwife is required and the salary offered. Any further particulars which may aid in the selection of a suitable candidate for the post should also be given.

### A GOOD START AT PORTSLADE.

It is less than a year ago since it was decided to secure a resident Queen's nurse for Portslade. The annual meeting has, in fact, been held at the expiration of nine months, and the chairman in his opening remarks said that the value of the nurse's services had been attested in the most enthusiastic manner. The most satisfactory feature of the favourable financial report, showing a balance of £22 on the side of income over expenditure, is the number of small subscriptions from persons unable to afford large amounts. During the nine months 2,719 visits were paid and 112 cases attended. This, as one of the speakers at the meeting suggested, indicates that a second nurse will soon be required, and it is therefore very important that the good start which has been made in Portslade should be speedily followed up by an extension of the list of subscribers, so that when the need becomes urgent there may be sufficient money in hand to justify her engagement.

### SHORT ITEMS.

The Incorporated Society of Trained Masseuses will hold a written examination on Tuesday, June 27th ; Anatomy and Practical on Friday and Saturday, July 7th and 8th, Monday and Tuesday, July 10th and 11th. Forms of application must be filled in and returned, not later than June 3rd, to the Secretary, 12 Buckingham Street, Strand, W.C. —In a recent issue of our Journal we referred to a Pocket Book for Midwives, published by Messrs. Allen and Hanbury, and quoted the price by a mistake as 1s. The book is supplied free on application.

### The Nursing Outlook.

"From magnanimity, all fear above;
From nobler recompense, above applause,
Which owes to man's short outlook all its charm."

### HOLIDAYS.

The time is approaching when everybody who can spare the time and the money is thinking of taking a holiday. There is no class of the community who earn their holidays harder than the residents in a hospital or than the large numbers who habitually labour amongst the sick. When at work their hours are longer, the conditions under which they labour expose them to many risks from which ordinary people are free, and the strain under which the work is habitually done tends to lower the system and render a good restful holiday and complete change essential to the continuance of the health and vigour of the worker. The probationer nurse whose friends reside in the country naturally spends her limited leisure with them during the holidays which fall to her share during the novitiate. The sisters and staff and private nurses who have completed their training, and whose earnings enable them to pay for a holiday, naturally desire to see something of the world, and to secure during their vacation as much change of scene and surroundings as their circumstances will permit. Formerly it was very difficult to secure a cheap holiday abroad, but nowadays many facilities are offered to the adventurous who wish to travel, or at least to see something of Continental life and scenery. With a view of facilitating the holiday arrangements of those who desire to travel, we have published for the last few years under the heading of "Travel Notes" many details which must have caused a number of our readers to desire a holiday on the Continent. The difficulty often is that a busy sister or nurse has little time or opportunity to spare for planning a tour, and to meet these cases inquiries addressed to our Travel Correspondent, stating exactly where the querist wishes to take her holiday, the amount of time available, and the approximate sum she is prepared to spend, are promptly dealt with.

We can well imagine, however, that the majority of nurses who aspire to travel may find it difficult to arrange for a suitable travelling companion, and may hesitate, unless they have a knowledge of French or German, to take their holidays abroad, however much the novelty of such an adventure may attract them. We recently gave particulars of Miss L. E. Walter's arrangements to conduct a party abroad, and now we learn that Miss L. M. Davidson, who is a near relative of a well-known sister in a great London hospital, is organising a party which she will personally conduct. Every detail is thought out and arranged beforehand, so that all the nurse has to do is to pay the inclusive sum which the trip will entail and to reach the rendezvous in time to join the party and accompany them abroad. Miss Davidson has arranged a tour for ladies only. The inclusive fee is 27 guineas, which includes all travelling expenses, hotels, tips, meals *en route*, and practically every other charge. Miss Davidson has a second tour leaving in August for 18 or 25 days, which includes Ober-Ammergau for the religious play, the "School of the Cross." The Bavarian Castle District will be visited, and will enable the party to see some of the finest dolomites and also the Rosenberg in Sud-Tyrol. The party travel second-class, which will be reserved, and the inclusive charge will be £22 10s. for 18 days, and £25 10s. for 25 days. It is proposed to visit the falls of the Rhine, and to include Lake Constance. These tours are specially arranged for gentlewomen who do not care to travel alone, and are glad to find pleasant companions for either long or short excursions (walking). Facilities are given for those who are adepts with the camera, and where possible arrangements are made to stay over Sunday at some place where there is an English service.

Those who do not care to go so far, and are fond of quiet, will find the Channel Islands, and especially Sark, a delightful and not too expensive place for a holiday. The Great Eastern and other railway companies publish a list of farmhouses and other accommodation, which give a wide range of choice at varying prices all over the country. The great point is to secure just that kind of life which offers the most attractions to the individual holiday-seeker, providing the maximum of restful change and comfort can be secured at a cost well within her means. Every worker needs a holiday which, by enabling some hobby to be pursued, will engage the attention and interest of the most worried of mortals, and secure that they forget all their worries and so give Nature a chance to recuperate from the ills which ambition or overwork or strain often produces in modern men and women. Nurses deserve well of the community and their employers, and we most certainly hope that the facilities we have afforded our readers to obtain the fullest information as to how and where to take a holiday may result in adding immeasurably to the happiness of the majority who have the energy and good sense to avail themselves of the opportunities placed at their command in our columns. We hope that those who are moved to do so may give us the benefit of any practical experiences, which might be useful to our Travel Correspondent, and spread a knowledge of the possibilities for beneficial change well within the reach of nurses. Thus much may be added to the happiness of some of the hardest worked and most deserving women workers in the world.

### The Nursing of Sick Children.

By James Burnet, M.A., M.B., M.R.C.P.Edin., Registrar, Royal Hospital for Sick Children; Clinical Tutor, Extramural Wards, Royal Infirmary; and Physician to the Marshall Street Dispensary, Edinburgh.

### VIII.—DISEASES OF THE NERVOUS SYSTEM.

The nervous system of the child is very unstable. It is readily excited and as readily depressed by very slight causes, causes which, as a rule, do not affect the adult in the very least. Disorders of the nervous system are accordingly commonly met with in young children. Infants and children so affected require a great deal of attention and the most careful possible nursing in order to avert those serious consequences which not infrequently supervene in such cases. In this lecture we shall consider two very common conditions of the nervous system, conditions which every nurse is bound to meet with at some time or other in the course of her professional duties. These two conditions are convulsions or fits, and meningitis or inflammation of the membrane covering the brain.

### Nature of Convulsions or Fits.

In many cases the attack is but slight and passes off in a few seconds. In these milder cases the child lies with eyes half closed, and presenting a somewhat fixed expression. The mouth twitches and the hands may be clenched with the thumbs buried in the palms. In the majority of instances, however, the convulsive seizure is of longer duration and of a much more serious nature. The patient often utters a cry and becomes immediately thereafter stiff and unconscious. The face is at first deadly pale, but presently becomes congested. The limbs are rigid, and the chest does not move with respiration. This is known as the tonic stage. Then comes the clonic or more truly convulsive stage in which the muscles of the face and jaws are moved into a variety of contractions, while the limbs are jerked about violently. The final stage is that of torpor in which the movements gradually cease, and the child falls into a more or less deep sleep.

### Some of the Commoner Causes of Fits.

Apart from true epilepsy these convulsive seizures may be due to improper feeding and to constipation. They may be brought on by fright, and may occur during the period of dentition. They are specially frequent in children who are the subjects of rickets. At the commencement of infectious diseases—such as measles and whooping-cough—fits are very frequently met with in young children, while pneumonia is sometimes ushered in by a convulsive seizure. Nurses should never accept teething as a satisfactory cause for fits. Healthy infants practically never have convulsions during the process of cutting their teeth, whereas unhealthy infants, and especially those who are the subjects of rickets, frequently do.

### The Value of Baths in the Treatment of Fits.

It is the usual custom to place every infant or child who has a fit into a bath of very hot water. This we consider not only unnecessary, but positively harmful in the majority of instances. The hot bath is given with a view to relieving the congestion of the brain which is supposed to be present; but in our opinion it not infrequently serves only to increase the congestion. Besides a hot bath often excites the little patient, and tends to bring on quite a succession of convulsive seizures.

### Cold should Always be Applied to the Head.

In order to reduce the congestion of the brain cold should always be applied to the head by means of an ice-cap, or, failing this, a handkerchief wrung out of cold water and frequently changed. At the same time the feet may be placed in a warm bath to which a little mustard has been added. In addition the physician may direct that a little chloroform be inhaled; but this, of course, is never left to the nurse. As a rule, it is advisable in every case to clear out the stomach and bowels. For this purpose the child should be given an emetic consisting of a teaspoonful of ipecacuanha wine, and at the same time a soap-and-water enema may be administered.

### Points which the Nurse Should Note Regarding a Fit.

The nurse should always write out a short report of every fit she has an opportunity of observing, with a view to giving information to the physician regarding its nature and duration. She should note how it begins, whether with or without a cry, and whether the convulsion is a general one, or whether only one part of the body, say an arm or leg, twitches during the attack. The nurse must also report whether consciousness is retained or lost, and how long the seizure lasts. Other facts to be noted are the presence of pallor or duskiness of the face, vomiting, and rise of temperature during the fit. If the patient has a succession of fits, the number and exact times at which these occur should always be carefully recorded for future reference.

### Causes of Meningitis.

Meningitis, or inflammation of the membrane covering the brain, may be brought about by quite a variety of conditions. It may be simple, in which case it may be due to invasion of this membrane by organisms met in the course of pneumonia, influenza, scarlet fever, middle ear disease, and conditions of a similar nature. In a great many cases, however, meningitis is tuberculous in character, a fact which renders it so commonly fatal in infants and children.

### Symptoms of Meningitis of Special Importance to the Nurse.

In every case of meningitis the nurse should carefully note the rate and general character of the pulse as regards its regularity and strength. The temperature is also of importance, though it is seldom very high, save towards the termination of the case, when it may rise to a considerable height. The respiration may be irregular and rapid, and in bad cases assumes a peculiar type known as Cheyne-Stokes. This form of breathing is readily recognised. At times the patient seems to have ceased breathing altogether, but presently the breathing is observed again, and it then becomes deeper and deeper until eventually it reaches a climax, when it immediately begins to die off again. This is usually a grave sign, and should receive special attention by the nurse in charge of the case.

Constipation is often a troublesome feature, and is usually best relieved by giving a soap-and-water enema every other morning. Vomiting may be fairly persistent, and the nurse is often greatly exercised in dealing with it. Bowel feeding may have to be resorted to, or even feeding by the nasal tube. We have, however, referred to these methods in a previous lecture so that further mention of them is unnecessary here.

When the patient becomes unconscious the bladder is apt to become distended, and accordingly the urine must be drawn off by means of a catheter. Towards the termination of the case the urine may be unconsciously passed by the patient, as may also the bowel contents. The child in this way is apt to be kept constantly wet and the bedclothes sodden. The nurse in these cases should endeavour to keep the patient as dry as possible by using sheets of cotton-wool, and applying plenty of powder about the thighs, groins, and buttocks.

Bedsores are readily produced in such cases, and they are best avoided by keeping the skin of the patient dry and clean. To this end methylated spirits should be painted over the lower part of the back and also over the buttocks. When bedsores form they must be treated like any other unhealthy wound.

Regarding the use of ice applied to the head a word of warning must be given. It may be used with advantage in a conscious patient with fairly high temperature and a tendency to fits. When, however, the child is unconscious or comatose, when the pulse is slow and irregular, and the breathing is also irregular in character, the use of an ice-bag is positively contraindicated. In many instances it brings about a state of sudden collapse, and therefore the nurse must never apply ice to the head on her own responsibility.

Meningitis of a tuberculous character is probably never recovered from, but this is no reason why the nurse should not exert herself to use every means to alleviate the patient's sufferings, and to satisfy the anxious parents and friends. She will then feel some satisfaction from the fact that she has done her duty, even if all her efforts have not availed to save the life of the little patient.

### The Nurses' Clinic.

### DIPHTHERIA IN DISTRICT NURSING. BY A SUPERINTENDENT OF DISTRICT NURSING.

Some few weeks ago a military doctor asked me to attend a case of diphtheria—the only son of an artilleryman. Married quarters usually consist of three rooms which, compared with those found in workmen's houses, are large and airy, but exceedingly bare and comfortless. The Government fittings consist of little more than a fixed wooden table and a few wooden seats without backs, and as a soldier rarely remains more than a year in one place and is only allowed 56 lbs. of luggage when he removes, it is impossible for him to accumulate much furniture.

Before entering the bedroom I asked the mother to send to the chemist for lint, 1d.; green protective or small piece of mackintosh, 2d.; absorbent wool, 1d.; bottle of collodion, 1d.; camel-hair brush, 1d.; bottle of carbolic (1–30), 3d.; half-ounce bicarbonate of soda; and ascertained that she had a warm piece of flannel out of which to make a binder.

I then took temperature, pulse, and respirations, and wrote a short report for the doctor. I washed the flanks well with hot water, soap, and methylated spirit, and put on a warm flannel binder. By this time the messenger had brought the lint, etc., and I made a carbolic compress of 1-60 by adding hot water to some of the 1-30 carbolic. I removed the very few useless articles from the room and knocked in a long nail on each side of the door, connecting them with a cord, from which I suspended a sheet dipped in 1-40 carbolic (commercial), a supply of which had been left at the quarters by the sanitary inspector.

I looked at the bare floor, the chilly whitewashed walls, the large bed far removed from the tiny fireplace, and asked the mother to try and borrow some screens or clothes-horses from the other married women, a chair-bedstead or flat sofa, a bronchitis kettle and trivet, some safety pins, and a few of the extra brown regulation blankets which Mr. Atkins at home uses—when he dares—for carpets. These were obtained, and left in the living-room until I was ready for them.

I covered the table with a clean newspaper, and arranged on it the articles that would be needed by the doctor:—

(1) A small clean enamelled saucepan, in which to boil the syringe and forceps; (2) a small carbolised bowl, in which to put syringe and forceps after they had been boiled; (3) a carbolised saucer with methylated spirit, in which to place needle; (4) collodion, brush, and a small piece of wool on a saucer; (5) a hand-basin with carbolic soap, nail-brush, and towels.

These preparations were just completed when the doctor arrived. I put a little cold water and half a teaspoonful of bicarbonate of soda into the saucepan, and boiled the syringe and forceps for seven minutes, lifting them out into the carbolised bowl, and placed the needle in a saucer with methylated spirit.

I then took the compress off, and the doctor injected the anti-toxin and sealed the puncture with collodion, and I put on warm wool and a binder. The doctor asked if we could not arrange some kind of tent. I brought in the chair-bed and placed it in front of the fire, placed screens on three sides of the bed, pinned the blankets all round, and put one on the top for a roof, turning back one small corner to allow the steam to escape. I half-filled the bronchitis-kettle and put it on the trivet to bring it a little further into the tent, hung up our own thermometer inside, and carefully explained to the mother that she must watch it and keep the temperature at 65° F. I asked her to keep a second kettle boiling, from which to supply the bronchitis-kettle, so that the air might be kept warm and moist. I wrapped a piece of warm flannel round the boy's throat, and asked the mother to give him five ounces of milk every two hours with soda-water, and to let him have strong beef-tea or chicken-broth four times in the 24 hours.

About 6 p.m. I made a second visit, and found that the doctor had been to see the child again in the interval, and had left orders that his throat was to be painted twice a day, and light hot fomentations applied. I explained to the mother that I should always need the same seven articles, and that it would be a great saving of time and trouble if she kept

### THE NURSES' CLINIC—*Continued.*

them together on a tray:—(1) A small tray lined with clean paper; (2) a glass of warm Condy; (3) a throat-brush or a piece of stick notched half an inch from the end and with a piece of lint fringed out and firmly tied at the notches, or two pairs of forceps with white wool to be twisted round; (4) an egg-cup in which to put the throat lotion; (5) a teaspoon to depress the tongue; (6) a bowl in which to receive expectoration.—N.B. Diphtheria patients must expectorate into carbolic 1-40, or perchloride 1-1000; (7) a towel to put round throat.

I asked the patient to lie down flat, and painted each side of the throat round quickly three times with glycerine and iron 1–4. I took temperature, etc., and bathed the hands and face.

The next day the doctor left instructions that the patient must be induced to gargle his throat with carbolic 1–40, and the treatment was varied by painting the throat with glycerine and borax, and light hot fomentations were applied to the throat.

The anti-toxin method was so successful that in a few days the membrane separated off the throat, and in a fortnight the child was convalescent. All discharges from the patient were placed in boiling water, and the lint brush for his throat was only used once and burnt immediately with the handle.

The diphtheria bacillus is to be found in the throat of patients months after their recovery, and as these germs (though harmless to the person himself) are a source of danger to others, I begged the mother not to allow the boy to go to school for at least three months, and during that time to make him gargle his throat three times a day with a glass of warm water to which a few drops of izal had been added, or with carbolic 1–80.

At the end of four weeks, finding there was no discharge from nose, ears, eyes, or throat, I obtained the doctor's permission to send for the sanitary inspector to have the room thoroughly disinfected. During the case I begged the mother to gargle her throat with Condy's fluid, at least thrice a day, to remove all rags with a pair of old scissors and burn them, to wash her hands with carbolic soap and water after performing all necessary offices, never to take her meals in the sick-room, and to banish the family cat, explaining that it could not only contract the disease, but could convey it to human beings.

The precautions necessary for the nurse are: Leave special sleeves and aprons at the house. Wash the hands well with carbolic soap and nail-brush, carbolise the thermometer. At the conclusion of her visit the nurse should go into the yard and gargle her throat with carbolic 1-80. After a short walk in the fresh air she should have a glass of milk and a biscuit.

Diphtheria occasionally causes paralysis, and massage and the use of the electric battery have to be resorted to. District nurses would do well to impress upon mothers the absolute necessity of attending at once to all complaints of sore throat. Recently I lost two diphtheria patients, a strong beautiful girl of thirteen and a child of four, the latter dying a horrible death of hæmorrhagic diphtheria. One had unquestionably been suffering for several days, and the other for an entire week before doctor or nurse were called in. In neither house was there real poverty, and in neither would such a degree of ignorance and callousness have seemed probable.

### Nursing in Italy and France.

### AN ENGLISH NURSE'S IMPRESSIONS.

NURSING on the Continent, especially in France and Italy, is gradually undergoing a very real and thorough transformation. For centuries the hospitals of these Roman Catholic countries have been dependent on the monastic institutions for their supply of sick nurses; and though some of these women have risen to the occasion most worthily, the greater number have performed their part in a spirit of blind obedience to the rules of their different orders, which is a very different thing from the obedience of trained intelligence and mental alertness such as we see exhibited in the bearing of the English hospital nurse of the present day. The nursing-nun is only answerable to the authorities of her order, who appoint her to certain duties without any reference to her aptitude or preference for those duties, and under such conditions an ideal nurse is hardly likely to be evolved. Her work must necessarily be mechanically performed through the deadening effect upon the mental faculties of her experience of monastic life. Yet such as it is and was, the nursing of the religious sisters was the only kind available until the latter part of the nineteenth century, when the influence of the work of Florence Nightingale began to make itself felt beyond the limits of Great Britain.

The rising tide of progress began in France when the foremost medical men in Paris realised that the nursing of the nuns in their great hospitals left much to be desired, limited as it was by their lack of training, and by the strict regulations imposed upon them by their spiritual superiors. Then commenced a battle which ended in the ejection of the religious sisters from the most important Paris hospitals, and a system of training was inaugurated which has done much to improve the nursing of the sick. This system starting with the principle that the probationer must begin her training as a maid-of-all-work, has the defect of only attracting young women of the servant class, and, naturally, these are handicapped from the beginning by the lack of a good education. The probationer is at first called a "fille de service," wears a special uniform, and takes no part in the nursing. After a certain period of probation she is promoted to be a "soignante," which really means a nurse. When her uniform is changed, and she attends lectures three times a week in a variety of subjects, such as physiology, anatomy, nervous diseases, children's ailments, fever nursing, etc., at the end of ten months she is subjected to a severe examination, and is then drafted off to another hospital where the training is on similar lines. If after some years' experience she is proved worthy, she is promoted to be a "surveillante," or sister of a ward, and then wears a black cap as a symbol of her promotion. A still higher dignity is possible, when she may be raised to the position of superintendent over several wards, which honour is symbolised by the wearing of a gold star in the front bow of her black cap. The uniform is a holland blouse and skirt and a white turn-down collar, with a large white apron It lacks the trimness of our English uniform, but could easily be transformed into business-like neatness by the touch of a trained matron.

This latter personage is unknown so far in the Paris hospitals, but changes are impending. The visit of the French medical men to London has not been without its effect; the nursing in our large hospitals created an impression which will not easily be effaced, and here I

may quote from a Paris nursing journal bearing date November 15th, 1904, in which a doctor says to the aspiring nurse: "Learn to be mistress of yourself; learn English self-control, so indispensable in life." The same journal also recommends to French nurses the desirability of getting in touch with professional subjects by reading systematically some British journal of nursing, by which method the French nurse may become better acquainted with the English language. These observations indicate the change which is gradually taking place in the minds of hospital authorities in Paris, and are very significant as to future probabilities.

Another important item is the translation of Miss Eva Lückes' "Manual for Nurses" into French. This work will be put into the hands of French nurses, and will be of especial value to them, because their instruction has so far been entirely in the hands of the doctors, and, as the aim of all hospital teaching is to place and keep the patient in the best possible condition whereby he may be cured, so the rôle of the nurse must be to suppress all sense of discomfort which is not a consequence of the patient's malady, and to be to some extent the moral support which every good nurse may be to helpless and suffering patients. This view of a nurse's rôle is being recognised more and more by the French doctors, and will doubtless have its influence in the alterations which are surely coming over the hospital nursing of France.

### The Nurses of Shoreditch Infirmary.

### INTERVIEW WITH THE MATRON. BY OUR COMMISSIONER.

Quite remarkable progress has been made at Shoreditch Poor-law Infirmary in the few years since it became a recognised training-school for nurses. At the recent examination the report subsequently sent by Mr. Stephen Paget to the Guardians shows that the standard of efficiency was very high. Another indication of the success of the training is that several appointments have lately been gained by nurses trained at Shoreditch. But before I asked Miss Joan Inglis, the matron, any details about the nursing on the occasion of my visit to the Infirmary the other day, she showed me over some of the wards of the building in Hoxton Street, and also the nurses' home.

Although it was not until 1899 that the training-school was established, and certificates issued at the expiration of three years, the Shoreditch Guardians have in one respect at any rate been pioneers of reform. They share with the Kensington Guardians the distinction of being the first Board in the metropolis to separate the workhouse from the infirmary.

"As to the training," said the matron, "the present system was originated when Dr. Norton was medical officer. He effected great improvements, especially in the maternity ward, which you have just seen, and our present medical officer, Dr. Froggatt, is still working improvements in that quarter."

"Then there was no trained matron before 1899?"

"The first trained matron only came in 1901, and I succeeded her in 1902. Previously I was matron of Leeds Union Infirmary, which has 800 beds, and is quite like a town within walls. But I desired to return to London."

"Were you trained in London?"

"Yes, at the London Hospital; and at the National Hospital for the Paralysed and Epileptic in Queen Square. I hold the massage and electrical certificates, and though I do not possess a midwifery certificate, I had obstetrical and gynæcological training at the London, and worked in those wards for 16 months. My first appointment in the provinces was that of night superintendent at the General Hospital, Wolverhampton, and I was then assistant matron and home sister at the Poplar and Stepney Sick Asylum, doing duty as matron in the absence of Miss Hannaford."

"How large is your existing staff?"

"Including nine sisters, sixty. There is one assistant matron, one home sister, and a night superintendent.

### Varied Training.

"And the mental wards?"

"The sister in charge of the mental patients has four female attendants under her."

"Your probationers, therefore, have the advantage of gaining experience in maternity and mental work as well as general nursing?"

"They all get a little of each; and, of course, it helps them afterwards."

"Do you use your up-to-date padded room frequently?"

"No, very seldom. Most of our patients are feeble-minded rather than violent."

"I suppose the probationers come on trial for a period?"

"For three months. When a candidate applies I always make private inquiries about her in order to ascertain whether she is suitable. At the end of three months it is quite easy for me to decide whether she has the making of a nurse. My opinion is that in that period a girl shows what she is made of. In these months she does bed-making, takes temperatures, learns how to wash helpless patients, in watching the cases to be observant. At the end of the time she does bandaging and general nursing. The second-year nurses go to the maternity ward for three months, the necessary number of cases being worked in during the three years' training."

"You mean that it has been the practice to obtain the certificate of the London Obstetrical Society?"

"Yes. Several have obtained that certificate. But we have not been recognised by the Central Midwives Board, though we have close upon a hundred cases during the year, and the wards are quite up to the standard. There is a maternity sister in charge and two nurses to assist her. I do not see any reason why we should not have been recognised before this. The application was made some time since."

### Lectures and Examinations.

"We were speaking," continued the matron, "of the second year's work, but I ought to have mentioned that in the first year the probationers have lectures on physiology and anatomy. In the second year I lecture on medical and surgical nursing, and on the nursing of fever; and in the third year the medical officer gives advanced lectures on medical and surgical work, including hygiene. A test examination is held after each course of lectures. The nurses take notes at each lecture, and do charts of cases. Note-books are supplied by the guardians for the probationers. Special attention is paid to writing and spelling. I am very particular that the handwriting should be legible, and the spelling accurate. At the end of three years an examination is held by Mr. Stephen Paget. As you know, the second has only just been held. Last year the nurses did very well, but Mr. Paget states that there was a great improvement this year. Practical classes are given by the assistant matron, and bandaging by the home sister, as well as by the ward sister."

"And all the nurses passed?"

"Yes, and some got 90 per cent. of marks. There were 10

### THE NURSES OF SHOREDITCH INFIRMARY—*Continued.*

candidates, and the examination was altogether a great success."

### Salaries and Off-Duty Hours.

"At what age do you admit candidates?"

"Between 21 and 30. They are provided with board, lodging, and washing, and, after their three months' trial, with indoor uniform. For the first year the salary is equivalent to £13 a year, the second year £18, and the third year £21. With the sanction of the Local Government Board, we are able at the end of 18 months, if they show capacity, to promote probationers to be staff nurses, with a salary of £25."

"How many beds are there in each of your airy wards?"

"As a rule, 72 on a floor. The sister-in-charge has two staff nurses and two probationers on the floor. At night there are one staff nurse and one probationer on duty. On the floor which includes the children's (18 in a ward), there is an extra probationer on duty during the day." The time off duty for all the staff is exceptionally liberal, in order that the nurses may get away from the surroundings of the Infirmary as much as possible. Thus the sisters have from 6 to 11 p.m. two evenings in the week, a half day weekly, 1 p.m. to 11 p.m., extended to a whole day monthly from 7 a.m. to 11 p.m. They also have leave every evening from 8 p.m. to

Some of the Nurses of Shoreditch Infirmary.
(*Photograph by Messrs. Gibbs and Co., Kingsland Road.*)

10 p.m., and they are away alternate Sundays from 3.15 p.m. to 11 p.m. The annual holiday is three weeks. There is not much difference between the off-duty time of the sisters and the staff nurses and probationers.

### A Liberal Diet.

"I noticed just outside your remarkably modern and well-kept kitchen, which is a great credit to the steward, that the diet for the nurses' dinner was posted up."

"The liberal diet is also a feature here. Indeed, I do not think that it could be improved upon."

"Might I have the bill of fare for the week?"

"Certainly."

"Monday: Roast beef, cauliflower, boiled potatoes, cornflour pudding (and jam). Tuesday: Roast mutton and onion sauce, greens, potatoes, Nelson pudding. Wednesday: Boiled ham, greens, potatoes, rhubarb pudding. Thursday: Soup; roast beef, Yorkshire pudding, cauliflower, baked potatoes, bananas. Friday: Fish and mutton chops, tomatoes, potatoes, jam tart. Saturday: Boiled beef and dumplings, carrots, potatoes, rice pudding, stewed figs. Sunday: Roast lamb and mint sauce, salad, mashed potatoes, rhubarb tart and custard.

All the meals are served in the sisters' and nurses' mess rooms in the infirmary.

### No Silence Rule.

"They are pleasant and commodious rooms. By the way have you a rule of silence at meals?"

"Indeed, we have not. I do not allow them to talk about the patients, but otherwise I encourage conversation at meals. I think it is most important for them to have recreation and other interests than their professional duties. They talk about the choir and about things in general. Our nurses come direct from their homes, and are quite as well educated as any at the London general hospitals. There is one point I emphasise when a new nurse comes, namely, that if she is unhappy or has any grievance, she can always come to me and I will do the best I can for her. I consider it very important that the probationers should all have fair play, and I go round the wards and see that they are properly instructed.

"You find it an advantage that your own quarters are in the Infirmary?"

"It is essential that they should be. Practically, as you can judge, I have a flat to myself, comprising offices, sitting-room, and sleeping accommodation."

"Was the Nurses' Home built before the school was established?"

"It was built two years earlier. There is a separate bedroom for every nurse."

"With a fireplace, I notice."

"Yes, I think it is most important to have a fireplace for the purpose of ventilation. There are hot-water pipes throughout the buildings and also the electric light. There is a separate sitting-room for the sisters and nurses, and a library. Breakfast is sent up to the nurses in their rooms on the mornings when they are off duty."

"You mentioned a choir. How long has it been in existence?"

"Over twelve months, and it consists of a score of nurses, who really sing splendidly. Nearly every Sunday we have an anthem, and I find that it helps to make the patients turn out to the service which is held in the large dining hall. I may mention that each nurse who is off-duty is permitted to attend her own church."

### Cookery and Uniform.

"Do you go in for cookery classes?"

"I am hoping to get some. The nursing should always come first, but the probationers ought to get a good knowledge of everything. I like the sisters to gain all the experience they can. In the summer holidays they come in for a share of general administrative work which helps them in obtaining higher appointments.

"Is the wearing of outdoor uniform optional?"

"Entirely. It is very nice for a nurse to have the cloak and bonnet to put on. But not a veil. I do not interfere with their costume so long as they dress nicely. The night nurses have a cycling club. Last year they had a tennis court at Victoria Park."

### The Successes at Shoreditch.

"I believe that your nurses have been singularly successful in securing outside appointments."

"Since 1902 three have become matrons, two superintendent nurses, one a home sister, and others who have left us are now ward sisters in London infirmaries, or charge nurses in the fever hospital. Some have gone to special hospitals to get more experience. I keep a record of every nurse after she leaves."

"In such circumstances, it is almost unnecessary to inquire if you have any difficulty in finding candidates for admission?"

"The only difficulty is that of selecting the best. There are about a hundred applications on the books at this moment."

### Select Committee of the House of Commons on Nursing.

Evidence of Sir Victor Horsley and Dr. Langley Browne.

The Select Committee of the House of Commons met again on Thursday last week. The evidence of Sir Victor Horsley, on behalf of the British Medical Association, and Dr. Langley Browne, of West Bromwich, which was substantially in agreement, was taken together.

Sir Victor Horsley said that the Association comprised close upon 20,000 medical practitioners, and that he was chairman of the representative meeting of the Association which met at Oxford last July. He quoted a resolution passed on that occasion to the effect that the meeting approved of the principle of the registration of nurses, while keeping an open mind on any Bills for registration which might be proposed. This resolution was, he said, carried *nem. con.*, the number of delegates being about 150 out of a possible 200. Sir Victor said that though one or two critical speeches were made against registration, the whole tone of the meeting was in favour of it.

With regard to revision of the register and deletion of names, asked by the chairman, Sir Victor said that he would not leave it in the power of a sub-committee to take away a nurse's calling from her. That ought to rest with the whole Council, but the sub-committee should establish a prima-facie case, just as with the General Medical Council. The sub-committee should act as a grand jury.

Sir Victor said it was his personal opinion that registration would not bring the nurse any more into competition with the doctor than she was at present, and that with the better-trained nurse the situation was less likely to occur. The remedy would lie in the hands of the Central Council. The contention that a certificate would "hall mark" a nurse applied equally, he said, to every professional man. If a nurse did not keep herself up to the general level of knowledge she would suffer in her work. Sir Victor did not believe that it would be difficult to secure a sufficient number of nurses. He thought that a fee of two guineas should be charged for registration and nothing for the examination. Dr. Langley Browne agreed that a large fee should not be required for registration.

Sir Victor thought that the registration of Institutions only would be of no value whatever. It would merely perpetuate the present system, which left too much power in the hands of an individual—namely, the head of the institution. The formation of a second class of registered nurses, to be called "nurse-helps," would, he thought, work unfavourably towards the trained nurse

Dr. Langley Browne said that the feeling in favour of registration was far stronger in the country than in London. With regard to nursing homes for patients, they were both of opinion that these should be registered and inspected, but that it was no use to register what might be called nursing hostels for the training of nurses if nurses themselves were on a State-register. They expressed themselves of opinion that greater uniformity of training was required and could only be gained by registration, and that three years were necessary to secure empirical experience to enable a nurse to attend any case.

Sir Victor urged that registration should be compulsory. There was some discussion as to what the term "compulsory" meant, and he explained that he thought it should be made illegal for any one to practise nursing for gain if not registered, after, say, five years. Sir Victor said he would define nursing as the responsible charge of a patient, and that the Court should decide the point on the merits of each case. If a woman were consistently looking after a patient in a house she would be really "nursing." No one would object, however, to any one, though untrained, putting on a poultice, for example.

Asked as to his opinion on the constitution of the Central Board, Sir Victor did not think that the lay element would be of much use. As to the examination, he said he knew of some examinations for nurses that were far too high, representing an amount of anatomical knowledge which no one could acquire without practising dissection. Others, no doubt, were equally too simple. He thought that the certificate should be simply one for nursing knowledge, with no reference to character.

Evidence of Dr. Bezley Thorne.

Dr. Thorne began his evidence by disclaiming that he was, as supposed, the prime mover in the demand of the Royal British Nurses' Association for registration. He had resigned the hon. secretaryship in 1895, and did not know beforehand that the question was being brought before the public. He had now resumed his work with the Association. He said he he did not agree with Sir Victor Horsley in his demand for universal registration. He thought that the time was not ripe to make it penal for anyone to nurse if not registered. As to revision of the register, he said that the British Nurses' Association revised its register, and that revision would be easier if it were a State register. He stated that 4,000 nurses had been registered by the Association, and that at present he should say there were nearly 3,000 on the register.

Evidence of Dr. Percy Allan.

Dr. Percy Allan, in his evidence, said that, from his experience as a general practitioner, he did not know how the poor were to be nursed if registration became compulsory. At present a patient would pay, say, 2s. 6d. for two nights for a woman to sit with her. Asked if the sick poor did not go to the hospitals, he replied that they frequently did, but often employed a woman who was not a trained nurse. If skill were required, the doctor would send the patient to a hospital. As to the possible danger in an unskilled person going to a case, Dr. Allan said there was a doctor in attendance, and he thought Sir Victor Horsley overlooked that point. As to the question of penalty, there was no penalty against anyone practising medicine—the only condition was that he must not call himself a doctor.

The Committee met again on Tuesday.

Evidence of Mrs. Hobhouse.

The first witness was Mrs. Charles Hobhouse, whose evidence was based on her experience in connection with the Rural Nurses' Association, Wiltshire, the Wiltshire County Midwives' Committee, and other associations in rural districts. She said that approximately a thousand nurses for rural and colonial purposes had passed through her hands during 12 years' work. She felt that any system of registration should consider the nurses fitted to nurse the sick poor in rural districts. Such nurses were generally not fully trained; in her experience she would say that perhaps the proportion would be one to eight, and that these not fully-trained nurses were quite satisfactory. The acute cases were sent to hospitals, and the best nurses would not remain in a rural district because of the lack of acute cases. Nor could rural districts support a fully-trained nurse. She had not found (as had been suggested) that a partially-trained nurse demanded just as high a fee as one fully trained. A highly-trained nurse was paid from £80 to £100, while partially-trained nurses earned from £20 to £50 with their board and lodging; there was no sort of competition between the two. Often these district nurses were first trained at the expense of an association at a training school, and then worked for the association. These schools, Miss Hobhouse explained later, practically took the place of a hospital ward; the nurses went out to patients, under the supervision of a sister, say for six months or a year. She feared that registration would be made too "close," but if it were reasonable she thought that it would be helpful. It would, she believed, give the provincial hospitals a status which they do not now hold and yet deserve.

Two guineas Mrs. Hobhouse considered a sufficient fee for both examination and registration, and she would charge a smaller fee to the less qualified nurses. She held that there must be two classes registered and two examinations.

With regard to the higher class, she laid more stress upon the curriculum undergone by a nurse than the period of training, and considered that two years was a sufficient minimum to insist upon. The training should be allowed in more than one hospital, so that hospitals should have power of co-operation. Registration should not be any guarantee of character.

For the lower class also she would have a curriculum and an examination. She considered that unless there were a lower class registered it would be hard for rural district associations to get nurses such as they were getting now. At present a nurse, after being with an association, would in many cases go to a hospital to train further, or would earn higher fees later by going to tradespeople and small farmers, where she generally earned, say 15s. a week. The tendency would be for all sorts of appointments to be given to fully-registered nurses only, and the number of registered nurses would increase. Thus there would be fewer future prospects to hold forth to the woman whom the association wished to train for rural district work. But if work done for a nursing association were considered a first instalment towards further training and in some way recognised, that would, she thought, meet her objection with respect to the shortage of rural district nurses which, she feared, would ensue from registration. As regards the poor, she thought they were sufficiently protected against bad nurses by the associations, but that registration would improve the status of nurses. She was strongly against any form of compulsory registration.

#### Evidence of Dr. Dickinson.

The next witness was Dr. Dickinson, who said that he had been in practice for 30 years in London and suburbs. He considered registration of nurses unnecessary, since he thought that the present arrangement was quite satisfactory; if the poor got bad nurses, it was the fault of the committee, and if the rich got bad nurses, it was their own fault. If nurses were registered, there would still have to be inquiry. As regards the finance of the matter, he was quite sure that any estimate based on a calculation that 60,000 nurses would pass on to the register would be altogether unsound—the number would be nearer 6,000. The General Medical Council had only enough money to go on with, and practically nearly every medical man was registered, and he felt sure the proposed Board would be short of money if the proposals in the Bills were carried out. He did not have any difficulty as a general rule in getting a suitable nurse; he relied entirely on the institution from whom he got her to make all inquiries. He thought that nursing homes and institutions which sent out nurses should be registered.

As to the resolution passed in favour of registration by the British Medical Association, he said, when asked by a member of the committee, that the Association comprised about half the number of existing medical practitioners, but that it was comparatively easy to get a resolution passed by the efforts of a few influential men; the general body of doctors were very apathetic, and the registration of nurses was not a burning question with them, though it might be considered to have awakened some interest.

#### Evidence of Miss Kent.

The evidence of Miss Kent, who said she had had a considerable nursing experience and was now doing private nursing independently, was then taken. She expressed her opinion that registration was most essential, and that it was considered so by the majority of nurses. She spoke strongly against the many small co-operative societies which had sprung up, which demanded as much as 25 per cent. of the nurses' earnings; they were often managed by people wholly unconnected with nursing, and she thought that a clause should be inserted in any Act to make it penal for anyone to manage such a society who had not some nursing qualification.

She was of opinion that the charge of the Central Midwives Board—one guinea for registration and 10s. 6d. for the examination—would be as much as a nurse could well afford.

### Central Midwives Board.

### SIR WILLIAM SINCLAIR'S MOTIONS.

A meeting of the Central Midwives Board was held at the Board-room last Friday. There were present Dr. Champneys (in the chair), Dr. Dakin, Miss R. Paget, Sir William Sinclair, and Mr. Parker Young.

A letter was read from the Clerk of the Council, transmitting an appeal for extension of time on behalf of a woman whose claim to be certified under Section 2 of the Act was made after March 31st, and another letter to the same effect from the Medical Officer of Health for Devonport. There was some discussion over this matter, Sir William Sinclair moving a resolution acceding to the proposed extension, but it was not seconded. Mr. Parker Young proposed that a reply regretting the Board's inability to comply with the suggestion be sent in both cases, and this was seconded by Dr. Dakin and carried. Sir William Sinclair then gave notice of a resolution calling attention to the hardship which would be dealt to many women in consequence of a rigid adherence to the letter of the law; he thought that some consideration should be shown to women who from ignorance had omitted to send in their applications in time. The Secretary was of opinion that the Board had no power to extend the time of grace without an amendment to the Act, which specified the date, and said that if one claim were admitted the same would follow in the case of some 200 women whose applications were sent in after March 31st.

The applications of 82 women for enrolment under section 2 of the Act were approved, bringing the total number now enrolled to 22,289.

The report of the Examination Committee was approved. The committee recommended the names of several examiners for addition to the list already approved, and further, that Dr. Champneys be appointed to assist the two examiners selected for the purpose of setting the paper at the first examination; also that a visitor be appointed to each of the provincial centres for the first examination, to report to the Board on the manner in which the examination was conducted, their necessary expenses to be defrayed by the Board. The Examination Committee consisted of Dr. Champneys, Dr. Dakin, and Mr. Parker Young.

The report of the Standing Committee followed, and was very nearly rejected, two members voting for and two against, the casting vote in favour being given by the chairman. The report dealt with various cases of alleged misconduct on the part of certified midwives. The applications for certification of three midwives were refused. The committee postponed several cases for further inquiry, and recommended that two certified midwives—accused, one of drunkenness, the other of non-compliance with the rules regarding the use of antiseptics, etc.—be cited to appear before the Board.

Cardiff Union Hospital, Croydon Union Infirmary, and West Ham Workhouse were approved as training schools, and nine medical practitioners were approved as teachers.

Sir William Sinclair then moved: "That the resolution of the Board of March 23rd, 1905, refusing the request of the Belfast Maternity Hospital for the recognition of its certificate as an approved qualification under Section 2 of the Midwives Act be rescinded." He contended that the Board was committing an illegal act in declining to recognise the Belfast Maternity Hospital, of which the application was in order, and the rights of the case clear. It was amazing that the Board did not see it in that light, but they had not heard the last of the matter. The resolution was not seconded and the matter dropped.

### Rural Midwives' Association.

THE ORGANISATION OF COUNTIES.

The second annual meeting of the Rural Midwives' Association was held at 66 Ennismore Gardens on Thursday afternoon last week. The chair was taken by Dr. Handfield Jones, and the report was read by Mrs. Heywood Johnstone, chairman of the Executive Committee. It states that the Association has during the past year sent 26 women to training institutions, making 51 in all since the Association was founded, and that of these 35 are now at work; 10 have not completed their training, four proved unsatisfactory, and two are refunding training-fees on plea of ill-health. The report also refers to a Training Home which Mrs. Johnstone is starting in a very populated part of London, where there is no maternity nursing going on. The report was adopted on the motion of Dr. Jones, seconded by Mrs. Murray. Certificates for good service were awarded to 17 acting midwives, these certificates being exchangeable at the end of three years for a certificate of merit.

The meeting then considered several points of difficulty which had arisen with regard to the working of the Midwives Act. The first question, namely, "the organisation of counties to cover the varying needs of small and sparsely-populated districts," was introduced by Mrs. Hobhouse, who said that it was quite impossible for such districts to stand alone. There must be a central organisation, and it should be based on the broadest possible lines in order to meet the varied systems that had been tried and found adequate for various needs. There were four grades of nurses to be remembered, the highly-trained who should work as a rule in the more thickly populated districts, village visiting-nurses for one village or group of villages, to go from house to house, cottage-resident nurses, and lastly those who acted as midwives only. It was necessary to find training vacancies, and it would be well to have a central home, not only for providing training but also for sending out nurses in cases of emergency. With regard to poor and less populated districts, villages might be grouped and together maintain one nurse, or, as had been successfully tried, a village might be affiliated to a larger association, and agree to employ a nurse for a certain number of weeks in the year at a time as required, say for 13 weeks at £12 a year.

Dr. Reid said that there were 1,200 midwives in his county (Staffs), of whom 500 were certificated. It was evident that before 1910, when the Act comes into full force, there would be a very great shortage throughout the country which would have to be supplied.

Mrs. Heywood Johnstone, speaking of the sort of woman the Association wished to become midwives, said that what they wanted was a woman who would turn her hand to anything, excepting, she hoped, washing clothes and scrubbing, and that general nursing seemed the best work to add to a midwife's own special work.

Dr. Handford, Medical Officer of Health for the Notts County Council, said that he did not think the cost of training was the greatest difficulty but rather the maintaining of a midwife after she was trained, and that to meet this local nursing associations would have to pay a midwife by salary, or the present dearth of candidates would continue.

Mrs. Heywood Johnstone then introduced the question of the payment of the doctor when called in by the midwife in accordance with the Act, and said she had forwarded to the Privy Council a resolution to the effect that the local Board of Guardians should be responsible for the fee to the doctor, which they should reclaim from those patients not under Poor-law relief. The charge to patients could be graduated according to their positions, as was done by some nursing associations.

Dr. Reid showed that the whole question involved more than the layman could settle. It touched medical etiquette. According to the Act, no doctor was obliged to attend in such cases, though he supposed that the meaning of the Act was almost tantamount to an obligation. There was further, the question: What doctor should be sent for? The Union Medical Officer should, he thought, be selected, and should be paid by the Board of Guardians who should recover from the patient where possible.

Mr. Hodgson, of the Cumberland County Council, said that he had been told that midwifery often formed the nucleus of a young doctor's practice, which would thus be taken out of his hands.

At the conclusion of the meeting Dr. Handfield Jones referred to the great loss the Association had sustained in the death of Mr. Heywood Johnstone, and congratulated the Association upon the work already accomplishied.

### Asylum Workers' Association.

THE PRIMATE'S SYMPATHY.

The annual meeting of the Asylum Workers' Association was held on Friday afternoon last, at the rooms of the Royal Medical and Chirurgical Society, Hanover Square. The chair was taken by Sir John Batty Tuke, M.P., president of the Association.

The report makes reference to the two Bills now before Parliament for the State Registration of Nurses, and also to the Bill to amend the Lunacy Acts, and in speaking of possible legislation in these matters, Sir John, who moved its adoption, said that the Medical Psychological Association had represented to the Select Committee that if the principle of the registration of nurses was agreed to by the committee, mental nurses should be included on the register. He used the term "mental" nurses, though he disliked the term immensely, for they all recognised that insanity was not a disease of the mind, but was caused by morbid corporeal conditions. If the principle of registration were agreed to the main difficulty would be to define the standard of registration. So far as existing asylum nurses were concerned, men as well as women, there would be no question, for the production of the certificate of the Medical Psychological Association, and evidence of having been for three years in one, or not more than two, asylums, and testimony of good character would be sufficient. But in the future he thought it extremely possible that another qualification would be asked for—i.e. training in a general hospital. He held that menta nursing was the highest order of nursing, demanding high moral qualities, patience, tact, and courage, and consideration for those under treatment. The addition of the technical knowledge, which could only be acquired in a general hospital, would be highly beneficial. As to the question of pensions for asylum officials which the Association had brought before the Government during the session 1904, Sir John pointed out that at the present time asylums were not allowed to grant pensions to their officers, and he knew of no class of public servants more deserving. He had no difficulty in promising that when the time came he would use what small influence he had to introduce a clause on this point in any amendment to the Lunacy Acts. He did not think that the time had yet come, for he held that the Bill now before Parliament was utterly inadequate, and he was doing his best to prevent it from reaching a second reading. He suggested that the need at the present time was the appointment of a Royal Commission to inquire into lunacy administration in Great Britain. The number of lunatics was increasing rapidly, and he considered that this was due to the accumulation of unrecovered patients, who would have recovered if they had been treated at an early stage of the disease. Moreover the inspection of asylums in England was utterly insufficient, and less than in Scotland. He had himself a bill before Parliament to grant London the same powers as are exercised in Glasgow, but it had no chance of passing, because of the opposition of the ratepayers. It seemed almost impossible to arouse public interest in the matter of lunacy, although it affected 4 per 1,000 of the community.

Sir James Crichton Browne seconded the adoption of the report, and congratulated the Association on its year's work. The Association was, he said, 3,000 strong, and had much influence.

The Archbishop of Canterbury said that the work in asylums had always been of interest to him. His first curacy had been at Dartford, and both here and at Stone, and in his subsequent charges, he had been brought in contact with some of the largest asylums in England, and had learnt to understand and respect the efforts of nurses, attendants, medical authorities, and chaplains, and he was glad to be able to express his sympathy to them that afternoon. There was an inexplicable lack of public interest as to what went on within the walls of our great asylums. He was glad to hear the Chairman speak of mental nursing as being, after all, the highest order of nursing. It needed moral powers of quite a different order from those called for in nursing patients who were sick and infirm. The public owed a great debt of gratitude to those who were carrying on the work, and he hoped that the utmost help would be given them.

Two gold and two silver medals were then awarded to four asylum workers, one a woman, for long and meritorious nursing service.

After discussion on the alteration of some minor points in the rules of the Associations, the question of the starting of a Mutual Benefit Fund for asylum workers was raised, but was referred to the Executive Committee.

Sir John Batty Tuke was re-elected President for the ensuing year.

### Where to Go.

Dowdeswell Galleries.—At these galleries, 60 New Bond Street, Mr. Maurice Greiffenhagen is exhibiting a collection of sketches of scenes in and near Naples. Mr. Greiffenhagen is a painter of originality, and on this occasion he has adopted a vigorous method to convey his impressions, which invests the exhibition with much individuality and distinction.

### Westminster Hospital Bazaar.

### THE NURSES' STALL.

The lofty towers of the Abbey looked down this week upon a unique and picturesque scene. Dean's Yard, where the bazaar was held for three days in aid of Westminster Hospital, was gay with coloured awnings, while the interior suggested an enchanted dream palace. There were 21 stalls, each represented by the costume of its holder some period in English history, from the time of the Conqueror to the reigning sovereign; they were prettily arranged in shades of green and old rose, the name of the holder and period being written in gold letters above.

The stalls formed a quadrangle, the space in the centre being occupied by the band-stand and the flower-stall under the management of the matron of Westminster Hospital and the nursing staff. It was a most picturesque centre-piece, laden with flowers and ferns of every variety; between arches of white trellis-work and palms trailing geraniums drooped. The nurses moved amongst the gay throng selling button-holes; some carried flat flower-baskets, suspended from the neck with scarlet ribbon; they wore uniform and, as flower vendors, looked irresistible.

One stall, representing James II., consisted of articles made by past and present patients of the hospital, was a substantial proof of their gratitude and appreciation. There were some beautiful pieces of needlework, prettily-dressed dolls, baskets, and metal work. The Hon. Mrs. George Greville and Mrs. Ritchie were the stall-holders. Mrs. Ritchie provided the patients with materials and has given an infinite amount of time and labour to make the stall a success.

The refreshment stall was managed by ladies of the medical and surgical staff of the hospital who brought back vividly the Commonwealth period, arrayed in the quaint Puritan style—dove-grey frocks and severe caps.

The bazaar was opened on Tuesday afternoon by the Duke of Connaught, accompanied by the Duchess, Princess Margaret and her royal fiancé. After the opening ceremony the royal party inspected the stalls, the society ladies, especially those who wore the Plantagenet headdress, looked like incarnated flowers, swaying in the wind, as they curtsied with old-fashioned grace to H.R.H. the Duchess, who afterwards visited and spent some little time at the nurses' stall. The stall representing George I. period was managed by Lady Thomas, assisted by Lady Hilda Murray, Miss Fanshawe, Mrs. and Miss Stockton, and Miss Armstrong. It contained articles for guilds, baskets and photogravures, it was a very interesting feature and the costumes worn by the ladies were charming.

Near the nurses' stall a celebrated Royal Academician in the guise of a pavement artist, worked, maimed for the occasion, surrounded by such notices as "Support Home Industries," "Please Contribute To The Poor Artist For The Orspital," while two top hats, one decorated with a yellow silk bow and labelled "For Gold," the other a modest grey colour, labelled "For Silver," left no doubt as to what manner of contribution was expected.

### To Nurses.

**We invite contributions from any of our readers, and shall be glad to pay for "Notes on News from the Nursing World," or for articles describing nursing experiences at home or abroad dealing with any nursing question from an original point of view according to length. The minimum payment is 5s. Contributions on topical subjects are specially welcome. Notices of appointments, letters, entertainments, presentations, and deaths are not paid for, but we are always glad to receive them. All rejected manuscripts are returned in due course, and all payments for manuscripts used are made as early as possible after the beginning of each quarter.**

### Everybody's Opinion.

[Correspondence on all subjects is invited, but we cannot in any way be responsible for the opinions expressed by our correspondents. No communication can be entertained if the name and address of the correspondent are not given as a guarantee of good faith, but not necessarily for publication. All correspondents should write on one side of the paper only.]

### CHILDREN'S HOSPITAL IN NEW SOUTH WALES.

"K. Ll. B." writes: Some few years ago I was asked to join a "Snowball" to collect used stamps for a Children's Hospital in Australia. The names of the hospital and of the town were told me; but Burdett's "Hospitals and Charities" had no mention of the hospital, and I believe the thing was a fraud. I should like to find out what was done with used stamps before collecting them for anyone.

### THE QUESTION OF A MATRON'S AUTHORITY.

"Provincial Matron" writes: With regard to the opinion which you express in favour of the decision of the managers of Swansea Hospital, "that it rests with the medical officer to decide whether a nurse is in a proper state of health to be on duty, and that when the medical officer decides that a nurse is not in a fit state for duty the matron should be immediately informed," does it not clash with your contention that, "the matron is the proper person to call to account for the conduct and condition of the nurses under her charge?" For my own part I endorse your contention, but object to the decision of the Swansea Board. I make it a rule myself that a sister should report to me the illness of any nurse working under her, and I think that if this is done, the medical officer ought not to interfere.

### REGISTRATION AND BADGES.

"Southbourne" writes: I do not think that either registration or badges are required for nurses. It rests with the general public. If they are particular only to employ nurses on the private staff of hospitals, or from *bona fide* institutions, I think there would be fewer complaints about nurses. I presume that in the latter case they should be allowed to see the history of the nurse, which should be fully entered in a book kept for that purpose, including length of training, certificates (if any), names of hospitals, etc., and length of time and reasons for leaving, or dismissal from, the last post where the nurse may have been employed up to the time of entering the institution. I am thankful to say that there are many conscientious women in the profession, but it must be remembered that there are good, bad, and indifferent nurses, as there are good, bad, and indifferent members of other professions. This always has been and always will be so. Registration or badges will not make nurses, however highly trained, any more conscientious. Let those who are nurses strive to be more worthy of their calling, less flighty and careless. We, who are constantly in the midst of suffering and death, ought to do our work from a very high motive.

### NURSES AND MONEY-MAKING INSTITUTIONS.

"Fair Play" writes: May I protest, in the name of common honesty, against the remarks made by "A Sympathiser" in last week's issue, *re* the keeping of agreements? If a nurse voluntarily joins an institution, and agrees to abide by the rules, even should she find a legal loophole of escape from her obligations, the moral obligation still remains. If a committee declined to pay "A Sympathiser" her salary when due, on the ground that their agreement with her was not binding, would she consider that fair? If a nurse objects to an agreement, why sign it? It is easy to refuse, but if she signs it is her duty to abide by it. If the writer looked at things from two points of view, instead of one, she would see that institutions cannot afford to give away their connection and their work to every nurse who wishes to get the advantages of having been in the institution, in order to benefit later by reaping where she has not sown. A nurse who recognises no obligations save those that can be legally enforced is not an enviable person, and unlucky indeed are those doctors and patients who may be putting their trust in her. Honesty, truth, and straightforward dealings are the very mainstay of all practical life, alike to employers and employed; fortunately most nurses recognise this, and play the game fairly. The small minority that prefers the lower levels of life may be disregarded; in the long run they will find themselves hopelessly outclassed.

### THE PARTIALLY-TRAINED NURSE.

"A Medical Nurse" writes: I have been much struck by some remarks ascribed to Sir Victor Horsley, in reference to the registration of nurses and the "ideal" state to which we might arrive when the untrained or partially-trained nurse would find it a penal offence to compete with her "registered" sister. It was borne in upon me what an "ideal" state of things that will be for the middle-class chronic patient, far more pitiable than the acute one, though such patient stands in as great need of good nursing; how could they afford the "registered" nurse during a protracted illness, even if the latest product of registration would condescend to nurse such an "uninteresting case"? When the "ideal" state is reached the doctor will no longer be allowed to provide the kindly woman fully competent to do such work. The "registered" nurse will have to be procured, or, failing that, the poor sufferer will have to be handed over to affectionate, but often ignorant, relatives. To the original disease will be added untold discomfort and suffering which the experiences of a good nurse would have spared the sufferer. If there were registration for medical, apart from surgical, nursing, the question would assume a different complexion. A woman who does her work faithfully and fully satisfies her patient, his friends, and the medical man, would not be afraid of submitting herself to an examination in practical nursing for medical cases. Such an one would not aspire to the rank of the "smart surgical nurse," the "blessed drudgery" of her profession quite satisfying her womanly instincts.

### Appointments.

[No charge is made for announcements under this head, and we are always glad to receive and publish appointments. The information, to insure accuracy, should be sent from the nurses themselves, and we cannot undertake to correct official announcements which may happen to be inaccurate. It is essential that in all cases the school of training should be given.]

Coventry City Hospital.—Miss M. Watson has been appointed charge nurse. She was trained at the Royal Free Hospital, London, and has since been sister at Bethnal Green Infirmary, charge nurse at the North-Western Fever Hospital, London, matron of Stoneyhurst College Infirmary, and matron of St. Anthony's College, Eastbourne.

Hertford General Infirmary.—Miss Eleanor Horsfall has been appointed head nurse, with charge of theatre. She was trained at Mill Road Infirmary, Liverpool, and at the Ladies' Charity and Lying-in Hospital, Liverpool. She has done private nursing, and is registered under the Central Midwives Board.

Military Families' Hospital, Colchester.—Miss Bessie Bristow has been appointed staff nurse. She was trained at the Miller Hospital, Greenwich, and is registered under the Central Midwives Board.

Mount Vernon Hospital for Consumption, Hampstead.—Miss F. M. Swain has been appointed matron. She was trained at St. Mary's Hospital, Paddington, and Bristol General Hospital. She has since been sister at Mount Vernon Hospital for Consumption, Hampstead, and matron of the Northwood Hospital from the time of the opening of that institution last summer.

Royal Hospital, Sheffield.—Miss A. Keeton has been appointed sister. She was trained at Sheffield Royal Infirmary, and has since been nurse at a private Ophthalmic Hospital in Sheffield.

St. Pancras Infirmary (South), Cook's Terrace, N.W.—Miss Ethel Gladys Derrers has been appointed holiday sister. She was trained at Southwark Infirmary, East Dulwich, and has since been ward sister at Bethnal Green Infirmary, and charge nurse at the Eastern Hospital, Homerton, N.

Salisbury Infirmary.—Miss Florence Freeman has been appointed sister. She was trained at Hull Royal Infirmary, and Queen Charlotte's Lying-in Hospital, London. She has since been staff nurse at the Cottage Hospital, Bromley, Kent, and sister at the Victoria Hospital, Blackpool.

Walton-on-Thames, Hersham, and Oatlands Cottage Hospital.—Miss Rosita Macandrew has been appointed matron. She was trained at the Hospital for Sick Children, Great Ormond Street, and at the Middlesex and King's College Hospitals, London. She has been nurse and assistant matron in connection with Queen Victoria's Jubilee Institute; has acted as locum tenens for the matron of the Livingstone Cottage Hospital, Dartmouth, and has taken charge temporarily of the Boys' Surgical and Convalescent Home, Banstead, Surrey.

Workhouse Infirmary, Norwich.—Miss Elizabeth Watson has been appointed charge nurse. She was trained at Liverpool Hospital for Cancer and Skin Diseases. She has since been charge nurse at the Union Hospital, Middlesbrough, night nurse at Dorchester County Hospital, and maternity nurse at Nottingham Union Hospital.

### Novelties for Nurses.

(By Our Shopping Correspondent.)

### THE HYGIENIC WARD SHOES.

The makers of these shoes, Messrs. R. Dick, 296 High Holborn, have sent a pair of these shoes for inspection. They are substantial and strongly made. The natural form of the foot is followed, the heel is of a sensible height, and the buttoned instep strap is an advantage. The price is moderate, and, with the attributes I have enumerated, the shoes will recommend themselves to nurses.

### ERASMIC SOAP.

Erasmic Soap has become very well known, and to those already acquainted with it it is unnecessary to recite its excellence. But to those who are yet strangers to it I can confidently recommend a trial. The purchase will not be regretted, as the soap is of very good quality, most delicately perfumed, and inexpensive. It can be obtained almost anywhere where soap is sold, or at the Company's depot, 117 Oxford Street, W.

### THE "EUREKA" CRÊPE VELPEAU BANDAGES.

These excellent bandages are in every respect as good as their inventor asserts them to be. They are good for all bandaging purposes, but will be sure to gain rapid popularity as an ideal article to be used for varicose veins in the legs. From the crêpé condition of the material, it gives to the form of the limb, without the necessity of spiral turns, and can be adjusted to supply accurately the amount of pressure required. An easily washed bandage for this purpose is most desirable, and Mr. Wood's invention offers no difficulty to the amateur laundress. The only precaution necessary is to lightly roll the bandage before drying, and to wring it across, not lengthwise. If this advice is adhered to, the material will retain its crêpé condition. The bandages are comparatively cheap, having regard to their eminently useful and durable qualities. They can be obtained from Mr. Vincent Wood, Victoria House, Albion Place, Blackfriars Bridge, S.E.

### Death in Our Ranks.

We regret to hear of the death of Miss Annie Hughes on Sunday last from typhoid fever, which she contracted while nursing at the Beaufort Isolation Hospital. She received her training at the Borough Hospital, Birkenhead, and had since been on the private staff of the Seafield Nursing Home, Cardiff.

### TRAVEL NOTES AND QUERIES.

By our Travel Correspondent.

Bruges or Brussels (M. A. G.)—If you go second class via Tower Bridge £1 1s. 7d. return, the £5 will last you a fortnight well and you can spend four days in Brussels, which is quite enough, and the rest at Bruges. At Brussels go to Hôtel de la Croisade, or to Hôtel du Rhin, 14 Rue de Brabant, ask for rooms on third or fourth floor, take coffee and rolls in the morning and late dinner in the hotel. Get lunch frugally wherever you are. Spend one day seeing Brussels itself, one visiting Mechlin, and one at Louvain. Then go to Bruges by an early train on the fourth morning. Stop at Ghent for a few hours, visit the town, and reach Bruges in the evening. Go to Hôtel Panier d'Or in the market place, spend nine clear days there, and you will return within the fortnight.

A Fortnight in Belgium for £10 (Queen's Nurse).—You can do it well as to money and time if you cut out Liège. That is too distant and not very interesting. Take this route. Antwerp via Harwich, second-class single, 15s. Stay three days there at Hôtel du Commerce, 10 Rue de la Bourse (rooms on third or four floor). Leave on fourth morning, visit Malines on the way to Brussels. At Brussels go to Hôtel de Bavière, stay there four days, visiting Louvain; leave on fifth day early for Ghent, seeing Alost on the way. At Ghent go to Hôtel aux Armes de Zeelande in the Marché aux Grains. Stay two days, on one of which visit Courtrai and Oudenarde. On the third morning go to Bruges and stay till your holiday is up at Hôtel Panier d'Or in the Market Place. Return to London by the G.S.N.C., second class 6s.

A Fortnight in Caen, etc. (E. B. M.).—Second-class return to Caen, via Havre, £1 11s. 8d. First class, £2 1s. 8d. Stay at Hôtel de Normandie. Madame Rivière will take you, I think, for 7 frs. per day, and I know you will be comfortable. But before settling down there I should spend three days in Lisieux; it is 30 miles further on. Go to Hôtel d'Espagne; always ask for rooms on third or fourth floor. There are charmingly picturesque streets and churches there, such as you wish to see. When at Caen, visit Bayeux one day, and another go to Dives and have tea at the Hôtel Guillaume le Conquérant; it is a typical old Normandy manor house. A third excursion quite easy from Caen is to Falaise. If you like to change your abode a third time go to St. Lô, about 40 miles south-west of Caen. Go to Hôtel Centrale, an interesting old town. From there a day's excursion will show you Coutances, with its splendid cathedral. If you want more help write again.

Brittany or Belgium (Stanley).—The amount you have at your disposal will not take you to Brittany for a fortnight, because the journey itself is expensive—£2 1s. 2d. You would enjoy Belgium quite as much. Do not go to cities, as you want fresh air especially. Your £6 will do a fortnight comfortably if you follow my directions. Return second-class fare to Ostend (via Tower Bridge) 9s.; second-class return to Namur, roughly speaking, £1. Then take steamer to Dinant; second return about 3s. —a lovely trip. Dinant is a beautiful place, full of cheap and easy excursions. Go to Hôtel des Familles, ask for rooms on fourth floor, and if they will take you for 6 frs. each for ten days or so? If, unfortunately, the house should be full, go on to Anseremme, a hamlet one mile distant, if possible still prettier. There go to Hôtel des Etrangers. Terms the same or rather less. If you stay a full fortnight it will rather strain your £6. It would be cheaper if you went only to Bruges and stayed with Mrs. Dear, Pension Internationale, 4 Rue Anglaise. Her terms are 5 frs. per day, and you would save the journey to Dinant, but from the point of view of health it would not be so good. Normandy is out of the question.

### Notes and Queries.

**For REGULATIONS see page 134.**

*Sanitary Inspector.*

(63) Can you kindly give me particulars as to how a nurse could become a sanitary inspector?—*Inquirer.*

Write to the Secretary, the Sanitary Institute, Margaret Street, W.

*Nursing Homes on the Continent.*

(64) Can you tell me the addresses of the nursing homes in Italy and the South of France and where to apply for information on district nursing?—*X. Y. Z.*

See "The Nursing Profession: How and Where to Train."

*Male Nurses.*

(65) I shall be very much obliged if you will kindly give me particulars for those desiring to become male nurses.—*W. H. W.*

The facilities open to men who desire to train as nurses are not very great in England. With the exception of the military and naval hospitals, only two or three schools exist for male nurses. Of these one is the National Hospital for the Paralysed and Epileptic, Queen Square, Bloomsbury, W.C.

*Epileptic Boy.*

(66) Will you kindly give me information with regard to a boy of nine suffering from epileptic fits. Is there a home for such cases free?—*H. G.*

It would be difficult to place a boy of this age in a free home. He had better try a hospital such as the National Hospital for the Paralysed and Epileptic, Queen Square, W.C., or apply to the National Association for the Employment of Epileptics, 12 Buckingham Street, Strand.

*India.*

(67) Will you kindly send me the address of "The Up-Country Nursing Association," as I wish for full particulars concerning it? *Emu.*

The address of The Up-Country Nursing Association for Europeans in India is Dalkeith House, Cambridge Park, Twickenham. Write to the Secretary.

*Tubing.*

(68) Can you advise me as to the kind of tubing I should get for an irrigator? The one in present use has perished very quickly.—*Inquirer.*

Messrs. W. H. Bailey and Son, 38 Oxford Street, London, W., would be able to advise you upon this point.

*Stewardess.*

(69) Would it be possible for someone to get a six months' training in any hospital to enable her to obtain a post as stewardess, which she could not do without at least six months' nursing experience?—*Irish.*

As a paying probationer six months' training can be obtained at several hospitals. See "The Nursing Profession: How and Where to Train."

*Training.*

(70) Is it possible to enter a large London training school for nursing after receiving a year's training in a small hospital? I feel that I want some such training prior to entering a large hospital, and if this be possible, could you kindly advise me as to where I should do best to apply?—*J. M.*

Unless you want to fill in an interval because you are too young to enter a large hospital, we should not advise you to have any prior training.

*Durban.*

(71) Will you kindly let me know your opinion as to the chances of two nurses going to East London, Durban, or Delagoa Bay, and where to apply as to posts of nursing, private, or otherwise?—*Nurse.*

The outlook for private nurses in South Africa is very dismal just now. See last week's HOSPITAL, which contained a warning on the subject written by a well-informed contributor. The Colonial Nursing Association, Imperial Institute, S.W., might advise you as to good openings in the Colonies. Write to the Secretary.

**Handbooks for Nurses.**

| | Post Free. |
|---|---|
| "How to Become a Nurse: How and Where to Train." | 2s. 4d. |
| "The Nurses' Dictionary" (Pronouncing) ... ... | 2s. 0d |
| "Nursing: its Theory and Practice" (Lewis.) ... ... | 3s. 6d. |
| "The Light Treatment" (just published) ... ... | 2s. 6d. |
| "A Complete Handbook of Midwifery." (Watson.) ... | 6s. 4d. |

Of all booksellers or of the Scientific Press, Limited, 28 & 29 Southampton Street, Strand, London, W.C.

### For Reading to the Sick.

### THE LOOM OF LIFE.

THE years of man are the looms of God, let down from the place of the sun,
Wherein we are weaving ever, till the mystic web is done.

Weaving blindly, but weaving surely, each for himself his fate;
We may not see how the right side looks, we can only weave and wait.

But, looking above for the pattern, no weaver hath need to fear.
Only let him look clear into Heaven—the Perfect Pattern is there.

If he keeps the face of the Saviour for ever and alway in sight,
His toil shall be sweeter than honey, his weaving is sure to be right.

And when the work is ended, and the web is turned and shown,
He shall hear the voice of the Master, it shall say to him, "Well done!"

And the white-winged angels of Heaven, to bear him thence, shall come down;
And God shall give him gold for his hire—not coin, but a glowing crown!

*Anson G. Chester.*

We are all of us like the weavers of the Gobelins, who following out the pattern of a well-known artist, endeavour to match the threads of divers colours on the wrong side of the woof, and do not see the result of their labours. It is only when the texture is complete that they can admire at their ease those lovely flowers and figures, those splendid pictures, worthy of the palaces of kings. So it is with us. We work, we suffer, and we see neither the end nor the fruit. But God sees it, and when He releases us from our task, He will disclose to our wondering gaze what He, the great artist, everywhere present and invisible, has woven out of those toils that now seem so sterile, and He will then deign to hang up, in His palace of gold, the flimsy web that we have spun.

*Frederic Ozonan.*

"There are no times in life when opportunity, the chance to be and do, gathers so richly about the soul as when it has to suffer. Then everything depends on whether the man turns to the lower or the higher helps. If he resorts to mere expedients and tricks, the opportunity is lost. He comes out no richer nor greater; nay, he comes out harder, poorer, smaller for his pain. But if he turns to God, the hour of suffering is the turning hour of his life. Opportunity opens before him as the ocean opens before one who sails out of a river. Men have done the best and worst, the noblest and the basest things the world has seen, under the pressure of excessive pain. Everything depended on whether they looked to the depths or to the hills for help."—*Phillips Brooks.*

"Let us take heed in time
That God may now be glorified in us:
And while we suffer, let us set our souls
To suffer perfectly: since this alone,
The suffering which is this world's special grace,
May here be perfected and left behind."

*E. H. King.*

## Figures and Tables

**Figure f1:**